# Comparative Study of Gabapentin, Clonidine and Placebo in Alleviating the Hemodynamic Changes Due to Tracheal Intubation and Laryngoscopy

**DOI:** 10.7759/cureus.37898

**Published:** 2023-04-20

**Authors:** Vikas Chauhan, Ajai Kumar

**Affiliations:** 1 Department of Anesthesiology, Columbia University Medical Center, New York, USA; 2 Department of Anesthesiology, Lady Hardinge Medical College, New Delhi, IND

**Keywords:** gabapentin, clonidine, laryngoscopy, tracheal intubation, premedication, hemodynamic changes

## Abstract

Introduction: Hemodynamic changes during laryngoscopy and tracheal intubation must be reduced for safe and effective anesthesia. The present study was conducted to compare the efficacy of oral clonidine, gabapentin and placebo in alleviating the hemodynamic changes due to tracheal intubation and laryngoscopy.

Methods: This was a double-blinded randomized controlled trial conducted on 90 patients who were undergoing elective surgery and were randomized into three groups. Group I (n=30) received a placebo, group II (n=30) received gabapentin and group III (n=30) received clonidine as premedication before anesthesia induction. Patient heart rate and pressor response were recorded periodically and compared between the groups.

Results: There was no significant difference in the baseline heart rate (HR) and mean arterial pressure (MAP) between the groups. HR elevation was observed in all three groups and found to be significant (p=0.0001) but the increase was higher in the placebo (15 min: 80.80± 15.41) and lower in the clonidine group (15 min: 65.53± 12.43). The elevation in systolic and diastolic blood pressure was least and transient in the gabapentin group, as compared to placebo and clonidine group. Intra-operatively, the requirement of opioids was higher in the placebo as compared to clonidine and gabapentin (p < .001).

Conclusion: Clonidine and gabapentin were effective in reducing the hemodynamic changes during laryngoscopy and intubation.

## Introduction

Direct laryngoscopy and endotracheal intubation during general anaesthesia can cause untoward responses in the human physiological system, especially in the respiratory and cardiovascular systems because of the noxious stimulus. Significant cardiovascular system changes like tachycardia, systolic and diastolic hypertension, a sudden increase in myocardial oxygen demand, and dysrhythmia can occur during laryngoscopy and endotracheal intubation [1]. These changes which occur are often related to the technique and duration of the procedure [2]. Even though, in healthy individuals, these responses are well tolerated and of transitory nature, untoward side effects like myocardial infarction, cardiac failure and hemorrhage can happen in coronary vascular disease and hypertensive patients. Ischaemic ST segment changes have also been reported during these procedures [3].

As both these procedures are key during general anesthesia, the hemodynamic responses that occur during the procedure must be alleviated to avoid untoward deleterious effects for the safe conduct of anaesthesia. Many different drugs and techniques have been used in previous studies with varying degrees of success to alleviate the cardiovascular response during these procedures [4-8].

Clonidine, a selective alpha 2 adrenergic agonist, alleviates the pressure changes during direct laryngoscopy and endotracheal intubation by its direct sympatholytic action. Clonidine reduces the arterial pressure through its action on both cardiac output and peripheral arterial resistance [9].

Gabapentin, a gamma amino-butyric acid neurotransmitter structural analogue has been used in the treatment of pain associated with neuropathy and as an anticonvulsant [10]. Gabapentin's effectiveness in alleviating the hemodynamic changes due to direct laryngoscopy and tracheal intubation has also been documented in a few studies, although the exact mechanism is still unknown. Possibly through its inhibition of voltage-gated calcium channels, gabapentin may cause this alleviation [10]. Hence, the present study aimed to evaluate the effectiveness of gabapentin and clonidine against placebo in alleviating the hemodynamic changes by tracheal intubation and laryngoscopy.

## Materials and methods

Study design

This double-blinded (study participant and outcome assessor) randomized controlled trial was carried out in Lady Hardinge Medical College, New Delhi between November 2009 and March 2011. In total, 90 patients, who were posted for elective surgery during the study period, were consequently enrolled, after getting permission from the institutional research and ethics committee (Lady Hardinge Medical College #IEC/2011/48).

Study participants included 90 patients above 20 years of age of both genders. Only American Society of Anesthesiologists (ASA) grading I and II patients were included in the study. Those with difficult intubation, emergency surgery, obesity, gastroesophageal reflux disease, drug allergy or systemic disease were the exclusion criteria for the study. Study participants were grouped randomly into three groups (Groups I, II and III) of 30 each. A simple randomization technique using a random number table generated from computer software was used. Routine care before anaesthesia was given similar to both groups of patients. All patients received tablet diazepam 5mg as a pre-anaesthetic medication on the previous day of intervention (12 hours before surgery).

Intervention

Two hours prior to administering general anaesthesia, trained health care personnel administered the study drugs to the patients. Group I received placebo, Group II received capsule gabapentin 900mg and Group III received tablet clonidine 0.2mg. As per the study protocol, the anaesthesiologist administering the general anaesthesia was blinded to the drug administered. Once in the operation room, baseline vital parameters were noted, followed by induction of anaesthesia. For induction, all patients received injection fentanyl, 1 μg/kg body weight i.v. and injection sodium thiopentone, 5mg/kg. To ease the procedure of laryngoscopy and tracheal intubation, muscle relaxant rocuronium bromide (1mg/kg) was administrated. 100% of oxygen was used during the induction of anaesthesia. The laryngoscopy and intubation were performed at 90 seconds after rocuronium administration by a well-trained anaesthesiologist. Oxygen and nitrous mixture at 33:66, rocuronium bromide and 1% sevoflurane were used thereafter for maintenance. Drop blood pressure was treated by optimizing the sevoflurane concentration and if needed boluses of vasopressor mephentermine bolus (5mg) were used. Any signs of inadequate analgesia i.e. tachycardia, hypertension and lacrimation, in spite of normal end-tidal carbon dioxide (EtCO2), were treated with an additional dose of i.v. fentanyl (1 μg/kg body weight).

Study variables

Patient heart rate, mean arterial pressure and blood pressure were recorded at baseline, before and after tracheal intubation and at regular intervals. Visual analogue scale in the immediate postoperative period was used in assessing the severity and duration of pain. The patients were followed up for the duration of their stay in the postoperative care unit, which was at least six hours.

Statistical analysis

Study variables were entered and statistically analysed in SPSS version 21.0 (IBM Corp., Armonk, NY, USA). Variables, which were continuous, were denoted in mean and standard deviation and categorical data were denoted in percentages. In order to determine the statistical significance between three continuous data, ANOVA was used. Chi square test was used to find statistical significance between categorical variables. Level of significance < 5% was used.

## Results

Average age and weight of the study participants were similar in all the groups. Although females dominated in all three groups, sex distribution was comparable. Laryngoscopy and tracheal intubation time duration, mean duration of surgery and rescue analgesia during anaesthesia were also comparable and statistically insignificant across all the study groups. Table 1 describes the study participant characteristics.

**Table 1:** Demographics and clinical characteristics of the study population *Denotes significant (P-value <0.05) NS=Not significant, P-value >0.05

Parameters (Mean ± SD )	Placebo	Gabapentin	Clonidine	P-value
Age (in years)	36.43±9.05	37.66±10.62	36.45±9.69	0.91^NS^
Weight (in Kg)	54.67±11.78	55.88±11.72	56.73±10.34	0.61^ NS^
Gender				
Female (n, %)	25 (83.3%)	25 (83.3%)	26 (86.6%)	0.79^ NS^
Male (n, %)	5 (16.7%)	5 (16.7%)	4 (13.4%)	
Duration of laryngoscopy and intubation (mins)	19.63±4.04	18.56±5.89	18.97±5.03	0.74^ NS^
Rescue analgesia during anaesthesia (mins)	1.61±0.56	1.02±0.00	1.04±0.30	0.16^ NS^
Duration of surgery (mins)	1.94±0.76	1.97±0.68	2.03±0.57	0.08^ NS^
Duration of post-operative analgesia				
0-2 hours	20 (66.7%)	1 (3.33%)	4 (13.3%)	0.01^*^
2-4 hours	10 (33.3%)	3 (10.0%)	4 (13.3%)	
4-6 hours	0 (0.00%)	6 (20.0%)	6 (20.0%)	
>6 hours	0 (0.00%)	20 (66.7%)	16 (53.34%)	

The heart rate values at the baseline were similar (P = 0.469) across the study groups. A highly significant heart rate elevation following direct laryngoscopy and endotracheal intubation was recorded in all three groups (P <0.0001), but heart rate rise duration was longest in the placebo group and of shortest duration in the clonidine group (Mean HR at 15 minutes: 80.80±15.41 in the placebo vs 65.53±12.43 in clonidine group). Table 2 describes the mean heart rate changes at various time charts.

**Table 2:** Mean heart rate changes at various time intervals among the groups *Denotes significant (P-value <0.05); NS=Not significant, P-value >0.05

Variables	Placebo	Gabapentin	Clonidine	P-value
Baseline	74.43± 10.81	75.70± 12.87	75.46± 13.03	0.469^NS^
Before Induction	75.56± 12.79	75.23±13.409	77.76±15.02	0.855^NS^
0 Minute	107.73± 13.86	90.90± 12.90	97.60± 13.97	0.0001*
1 Minute	102.43± 14.69	86.133± 13.17	93.46± 14.98	0.0001*
3 Minute	91.86± 12.23	83.46± 12.08	77.43± 11.36	0.0001*
5 Minute	88.20± 15.32	74.53± 12.47	74.86± 14.67	0.0001*
10 Minute	83.33± 13.50	71.30± 12.79	69.60± 13.00	0.0001*
15 Minute	80.80± 15.41	70.26± 13.34	65.53± 12.43	0.0001*

The mean systolic blood pressure measured at baseline was similar across the study groups (P = 0.557). Increase in systolic blood pressure was least and transient in patients who received gabapentin, in comparison with the placebo and clonidine group. This difference noted was statistically significant (P <0.0001). Table 3 describes the mean systolic blood pressure changes at various time charts.

**Table 3:** Mean systolic blood pressure changes at various time intervals among the groups *Denotes significant (P-value <0.05); NS=Not significant, P-value >0.05

Variable	Placebo	Gabapentin	Clonidine	P-value
Baseline	126.1± 12.908	124.471± 14.312	125.433± 13.272	0.557^NS^
Before Induction	124.06± 14.152	124.233± 15.328	124.466± 14.805	0.908 ^NS^
0 Minute	147±15.754	134.366± 13.943	137.0± 14.023	0.0001*
1 Minute	146.776± 15.887	122.723± 15.711	132.665± 14.763	0.0001*
3 Minutes	136.133± 14.032	107.966± 15.089	119.5± 14.577	0.0001*
5 Minutes	127.5± 14.265	107.80± 10.468	116.3± 16.613	0.0001*
10 Minutes	120.533± 12.819	106.433± 12.991	113.666± 15.827	0.0577^NS^
15 Minutes	120.866± 13.441	104.80± 15.359	112.623± 14.545	0.0308^ NS^

Study groups' baseline mean diastolic blood pressure was found similar (P = 0.428). The gabapentin group reported minimal elevation in mean diastolic blood pressure and was also found to be transient. The placebo group had elevated diastolic blood pressure for longer duration (P <0.0001). Table 4 describes the mean diastolic pressure changes at various time charts.

**Table 4:** Mean diastolic pressure changes at various time intervals among the groups *Denotes significant (P-value <0.05); NS=Not significant, P-value >0.05

Parameters	Placebo	Gabapentin	Clonidine	P-value
Baseline	80.20± 10.012	80.411± 12.913	81.432± 11.081	0.428^NS^
Before Induction	81.11± 12.01	82.116± 11.819	80.119± 12.918	0.439^NS^
0 Minute	99.68± 13.281	86.422± 12.08	91.441± 10.681	0.0001*
1 Minute	96.913± 11.91	77.113±10.281	87.81± 12.289	0.0001*
3 Minutes	90.681± 10.08	66.231± 13.11	78.28± 11.89	0.0164*
5 Minutes	87.11±12.11	66.11±10.821	76.11± 13.049	0.0008*
10 Minutes	86.11±11.91	65.33±11.88	74.43± 12.81	0.161^NS^
15 Minutes	85.11±13.81	65.42±12.81	73.10±11.60	0.290^NS^

There was not much difference found in the mean arterial pressure (MAP) baseline values (P = 0.781). The placebo group had a higher and longer duration of increase in the values compared to other groups. (Mean MAP at 15 minutes: 96.002±11.87 in the placebo vs 78.88±-13.03 gabapentin group) (P <0.0001). Table 5 describes the mean diastolic pressure changes at various time charts. Intraoperative opioid drug requirement was high in the placebo group in comparison with other groups (P <.001). Intraoperative analgesia was given using injection fentanyl 1 μg/kg in our study. As already shown in Table 1, clonidine and gabapentin groups had reduced requirements of intraoperative analgesia compared to placebo groups.

**Table 5:** Mean arterial pressure changes at various time intervals among the groups *Denotes significant (P-value <0.05); NS=Not significant, P-value >0.05

Parameters	Placebo	Gabapentin	Clonidine	P-value
Baseline	95.60±11.81	95.01±12.02	96.65±10.66	0.781^NS^
Before Induction	95.88±12.99	94.13±10.27	95.76±11.87	0.901^NS^
0 Minute	115.42±10.22	102.89±11.23	106.75±12.98	0.0001*
1 Minute	112.32±12.67	92.90±12.83	103.01±10.67	0.0001*
3 Minutes	105.68±11.33	79.88±10.87	91.89±11.32	0.0001*
5 Minutes	100.02±10.79	79.44±10.09	89.86±12.99	0.0001*
10 Minutes	97.88±13.33	78.90±11.33	87.93±10.76	0.0003*
15 Minutes	96.00±11.87	78.88±13.03	86.77±10.78	0.0001*

## Discussion

This study was carried out to compare the effectiveness of gabapentin and clonidine when given separately with placebo in alleviating the hemodynamic changes due to direct laryngoscopy and endotracheal intubation.

Though the hemodynamic changes due to direct laryngoscopy and endotracheal intubation are usually tolerated well in healthy individuals, the cardiovascular response can cause catastrophic effects in patients with coronary vascular disease [1]. Sudden release of catecholamines including norepinephrine, epinephrine and vasopressin can cause hypertension and an increase in heart rate. Sudden rise in intracranial pressure has also been reported. Therefore, the pressure response which occurs during tracheal intubation and laryngoscopy should be alleviated to minimize the risk of catastrophic events in those patients. Premedication is frequently used during the practice of anesthesia and an ideal premedication should be effective in maintaining cardiovascular stability without any significant side effects.

Our study reported that both gabapentin and clonidine when given separately were able to alleviate the heart rate increase following direct laryngoscopy and endotracheal intubation but not completely. The placebo group had sustained elevation of heart rate in comparison with others.

Singhal et al. [11] reported less rise in heart rate after laryngoscopy and tracheal intubation after patients received clonidine as a premedication. Also, the clonidine group reported transient elevation of heart rate of 5.55% after the procedure.

Memis et al. [12] found that gabapentin premedication resulted in less elevation of heart rate values after the procedure in comparison with placebo. A statistically significant difference (P <0.0001) in heart rate at various time duration was reported after administration of gabapentin as a premedication.

However, Fassoulki et al. [13] reported no statistical difference in increase of heart rate following the procedure after gabapentin administration as compared to placebo. The study reported that the mean heart rate was similar between the gabapentin and placebo measured at various time intervals after the procedure.

Another study also reported that, though gabapentin was not able to completely alleviate the heart rate after direct laryngoscopy and endotracheal intubation, it was better than placebo [14].

The pattern of increase in systolic, diastolic and mean arterial pressure among the three groups following direct laryngoscopy and endotracheal intubation were reported to be similar. Gabapentin was highly effective in alleviating the pressor response, followed by clonidine in comparison with the placebo group. The fall in MAP below baseline value in the gabapentin and clonidine groups after one and three minutes following laryngoscopy and intubation is probably because of the effect of anaesthesia drugs and absence of surgical stimulus until 15 minutes of observation, which came back to normal after surgical stimulus without any intervention. Our results were similar to the study done by Marashi et al. [14] which found a similar pattern in pressure response between the three groups. They also reported that both systolic and diastolic blood pressure significantly differed, with the highest values being noted in the placebo and the lowest in the gabapentin.

Similar kinds of results have been reported in many previous studies [15,16]. Kamran et al. [17] reported that systolic arterial pressure (SAP), diastolic arterial pressure (DAP), and MAP measured at various time duration after the procedure were significantly lower in the gabapentin group, in comparison with clonidine (P <0.05).

Intraoperative analgesia was given using injection fentanyl 1 μg/kg in our study. Clonidine and gabapentin groups had reduced requirement of analgesia compared to placebo groups. Pandey et al. also found that patients who had gabapentin as a premedication reported lower pain scores at all times postoperatively in comparison with placebo [18]. Similar reduction in opioid consumption during the perioperative period in patients who received gabapentin and clonidine pre-operatively has been reported in other studies [15,19].

Limitations of the study

This study was conducted with a limited number of participants in each group. Patients with ASA physical status I and II were enrolled in the study, so the results cannot be generalized to the patients with higher ASA status. Ideally, a large multi-centre study involving a large number of participants is required to establish the effectiveness.

## Conclusions

Oral administration of both clonidine and gabapentin was effective in alleviating heart rate and pressor response (SBP, DBP and MAP) during laryngoscopy and endotracheal intubation. Intraoperative analgesic requirement was reduced and good postoperative analgesia was observed in both clonidine and gabapentin as compared to placebo.
